# Ultrasound combined with glial cell line-derived neurotrophic factor-loaded microbubbles for the targeted treatment of drug addiction

**DOI:** 10.3389/fbioe.2022.961728

**Published:** 2022-08-15

**Authors:** Feng Wang, Hongwei Wu, Azhen Hu, Lei Dong, Xiaoxia Lin, Menghao Li, Yongling Wang, Wenjun Li, Liansheng Chang, Yuqiao Chang, Hanqing Liu, Yu Shi, Nana Li

**Affiliations:** ^1^ Henan Key Laboratory of Medical Tissue Regeneration, School of Basic Medical Sciences, Xinxiang Medical University, Xinxiang, Henan, China; ^2^ Department of Ultrasound, Peking University Shenzhen Hospital, Shenzhen, China; ^3^ Department of Chemistry, Xinxiang Medical University, Xinxiang, Henan, China; ^4^ Shenzhen PKU-HKUST Medical Center, Shenzhen, China; ^5^ Department of Physiology and Pathophysiology, School of Basic Medical Sciences, Xinxiang Medical University, Xinxiang, Henan, China; ^6^ Division of Oral and Craniofacial Biomedicine, University of North Carolina Adams School of Dentistry, Chapel Hill, NC, United States; ^7^ Department of Human Anatomy Histology and Embryology, School of Basic Medical Sciences, Xinxiang Medical University, Xinxiang, Henan, China; ^8^ Central Laboratory, Shenzhen Samii Medical Center, Shenzhen, Guangdong, China

**Keywords:** focused ultrasound, targeted microbubbles, blood-brain barrier, drug addiction, glial cell line-derived neurotrophic factor

## Abstract

Drug addiction is a serious problem globally, recently exacerbated by the COVID-19 pandemic. Glial cell-derived neurotrophic factor (GDNF) is considered a potentially effective strategy for the treatment of addiction. Previous animal experiments have proven that GDNF has a good therapeutic effect on drug addiction, but its clinical application is limited due to its poor blood-brain barrier (BBB) permeability. Low-frequency focused ultrasound, combined with microbubbles, is a non-invasive and reversible technique for locally-targeted BBB opening. In the present study, magnetic resonance imaging-guided low-frequency focused ultrasound, combined with GDNF microbubbles, was used to target BBB opening in the ventral tegmental area (VTA) region. The effects of GDNF on morphine-induced conditioned place preference (CPP) and acute withdrawal symptoms in rats after a partially opened BBB were evaluated by behavioral observation. Western blot was used to detect changes in tyrosine hydroxylase (TH) expression levels in the VTA region after different treatments, and high performance liquid chromatography was used to detect the changes in monoamine neurotransmitter content. The results showed that ultrasound combined with GDNF microbubbles targeted and opened the BBB in the VTA region, and significantly increased GDNF content, destroyed morphine-induced CPP, and reduced the withdrawal symptoms of morphine addiction in rats. Furthermore, the up-regulation of TH expression and the increase of norepinephrine and dopamine content induced by morphine were significantly reversed, and the increase of 5-hydroxytryptamine content was partially reversed. Therefore, ultrasound combined with GDNF microbubbles to target and open the BBB can effectively increase the content of central GDNF, thus playing a therapeutic role in morphine addiction. Our study provides a new approach to locally open the BBB and target delivery of neurotrophic factors, such as GDNF, to treat brain diseases like addiction.

## Introduction

Drug addiction is a serious problem worldwide (Allen et al., 2013). According to the World Drug Report 2021 (Becker and Fiellin, 2020), published by the United Nations Office on Drugs and Crime, approximately 275 million people used drugs in 2020, an increase of 22% from 2010, and more than 36 million people develop mental disorders as a result of drug abuse. By 2030, demographic factors project the number of people using drugs will increase by 11% worldwide. Drug use killed almost half a million people in 2019, while drug use disorders resulted in 18 million years of healthy life lost, mostly due to opioids. The COVID-19 pandemic has further exacerbated the drug problem by creating conditions that make more people susceptible to drug use and to engaging in illicit crop cultivation. For example, the COVID-19 crisis pushed more than 100 million people into extreme poverty, with 114 million jobs lost globally in 2020. In contrast, drug traffickers have quickly recovered from the initial setback caused by lockdown restrictions and are operating at pre-pandemic levels. Furthermore, access to drugs has become simpler than ever, with an increase in online sales and contactless drug transactions, such as by mail, a trend possibly accelerated by the pandemic.

Opioid drugs are among the most powerful analgesics but also among the most addictive. Opioids are the most widely used addictive substances with the longest history (Belin et al., 2007). According to China’s 2020 drug situation report, by the end of 2020, China had 1.801 million drug users, 734,000 of whom abused opioids, accounting for 40.8% of the total number. In the United States, approximately 50,000 people died from an opioid overdose in 2019, more than double the number of deaths compared to 2010. North America has seen a spike in opioid overdose deaths since the onset of the pandemic. For example, opioid overdose deaths in Canada were 58% higher during the April–June 2020 quarter compared with the same period in 2019. COVID-19 could lead to infection in persons with opioid use disorder, higher opioid overdose rates, reversal of system-level gains in expanding access to medications for opioid use disorder, halt critical research, and prevent exacting legal reparations against opioid manufacturers (Bleier et al., 2016). These could cause an intensification of the opioid crisis.

Understanding the mechanisms of opioid addiction and finding treatment methods have been goals of scientists and doctors for more than a century. Since the mid-1990s, neurotrophic factors, such as brain-derived neurotrophic factor (BDNF) and glial cell line-derived neurotrophic factor (GDNF) have been receiving much attention, especially in drug reward and relapse (Carnicella et al., 2008). GDNF is a neurotrophic factor that promotes the survival of embryonic midbrain dopaminergic neurons. Accumulating evidence suggests that GDNF plays a unique role in negatively regulating the actions of drugs of abuse; thus, the GDNF pathway may be a promising target for the treatment of addiction (Carnicella and Ron, 2009). However, the large molecular weight of GDNF (24 kDa) makes it difficult for it to cross the blood-brain barrier (BBB), which limits its application in disease treatment (Chen et al., 2020). Therefore, how to promote the transmission of GDNF through the BBB and increase the effective drug concentration of GDNF in the central nervous system (CNS) is a key scientific problem for the application of GDNF in the treatment of drug addiction.

In fact, the BBB is one of the major factors restricting the use of many CNS medications (Cheron and Kerchoved'Exaerde, 2021). In recent years, scientists have tried many ways to promote the transmission of GDNF through the BBB, such as viruses (Cintrón-Colón et al., 2020), carrier nanoparticles (Everitt and Robbins, 2016), fusion proteins (Fu et al., 2010), and heterotopic mucosal grafts (Gasca-Salas et al., 2021). Focused ultrasound-induced BBB opening is considered a non-viral, non-invasive, and targeted method for drug and gene delivery (Ghitza et al., 2010; Holt, 2021). Focusing ultrasound to open the BBB and increase intracerebral delivery of GDNF has been proven to be safe and effective, and has shown good application prospects in the treatment of a variety of diseases (Kastin et al., 2003; Kilic et al., 2003).

In our previous study, magnetic resonance image (MRI)-guided focused ultrasound was proven to successfully open the BBB and achieve the targeted delivery of GDNF (Lin et al., 2016). Furthermore, we developed GDNF-loaded microbubbles (MBs) and achieved local and precise delivery of GDNF to the CNS through MRI-guided focused ultrasound-induced BBB disruption, and confirmed its therapeutic effects on chronic mild stress rat model of depression (Lin et al., 2015). However, MRI-guided focused ultrasound combined with targeted MBs to open the BBB and deliver GDNF has not been reported for the treatment of drug addiction. Therefore, the present study aimed to use MRI-guided focused ultrasound combined with targeted MBs to open the BBB, promote GDNF crossing the BBB, and target delivery to the VTA region. A morphine addiction model was established, and behavioral observation was used to evaluate the effects of GDNF on acute withdrawal symptoms and psychological craving in rats after partial opening of the BBB. Concurrently, high performance liquid chromatography (HPLC) was used to determine the content of monoamine neurotransmitters in the brain, and to measure the effects of increased brain GDNF levels on central monoamine neurotransmitters after partial opening of the BBB. These results will help determine the target and mechanism of GDNF for detoxification, and provide a scientific basis for the use of GDNF across the BBB for the treatment of drug addiction.

## Materials and methods

### Preparation and characterization of GDNF-Loaded microbbules

In this study, MBs were biotinylated and lipid coated, which encapsulated a high-molecular-weight gas core of perfluoropropane (C3F8) (Lin et al., 2019). Before preparing the drug-loaded MBs, the MBs were washed three times in a phosphate-buffered sodium (PBS) solution to remove the excess unreacted lipids by centrifugation at 400 *g* for 3 min. A specific amount of avidin (final concentration 3 mg/ml) per 10^9^ MBs was then added to the washed MB dispersion. After incubating at room temperature for 15 min, the MBs were washed three times to remove the unreacted avidin further and then incubated with 200 μL biotinylated GDNF. The average diameters of the plain control and GDNF-loaded MBs were determined by an Accusizer (Model 780A; Particle Sizing System, Santa Barbara, CA, United States).

### Animals

Male Sprague–Dawley rats (weighing 200–220 g upon arrival) were provided by Guangdong Medical Laboratory Animal Center (Foshan, Guangdong, China) and housed five per cage (before stereotactic nucleus localization surgery) or individually (after stereotactic nucleus localization surgery) under a 12-h light/dark cycle at 22 ± 2°C and relative humidity of 50% ± 10% with *ad libitum* access to food and water. The animals were allowed to acclimatize to a specific pathogen-free environment for 7 days prior to treatment. This animal study was performed in Shenzhen PKU-HKUST Medical Center (animal license number: SYXK 2015-0106) and approved by the Ethics Committee of Peking University Shenzhen Hospital. All of the animal procedures were performed in accordance with the National Institutes of Health Guide for the Care and Use of Laboratory Animals, and the procedures were approved by the Local Animal Use Committee.

### Conditioned place preference

The conditioned place preference (CPP) procedure was performed in a three-chamber apparatus using an unbiased, counterbalanced protocol as described previously (Lin et al., 2020). A brief description of the procedure is as follows. Baseline preference was assessed by placing the rats in the center chamber of the CPP apparatus and allowing them to explore all three chambers freely for 15 min. Rats that showed a strong unconditioned preference for either of the side chambers (i.e., >540 s) were excluded from the experiments. The remaining rats were then trained for eight consecutive days with alternating subcutaneous injections of morphine (10 mg/kg) or saline (1 ml/kg) and were confined to the conditioning chambers for 45 min after each injection. The test for the expression of methamphetamine-induced CPP was identical to the initial baseline preference assessment and was performed on the following day after training.

### Stereotactic brain surgery

The rats were anesthetized with intraperitoneal (IP) pentobarbital sodium (50 mg/kg) and placed in a stereotaxic apparatus (RWD Life Science Co., LTD., China). The skull was exposed and a cannula was inserted 1 mm above the target brain region (ventral tegmental area, VTA) at the following coordinates (Lin et al., 2019): anterior/posterior (AP), −5.2 mm; medial/lateral (ML), ± 1.8 mm; and dorsal/ventral (DV), −7.5 mm. The cannula was secured to the skull with anchoring screws and dental acrylic cement. A stainless-steel probe was inserted into the cannula to maintain patency, and penicillin (200 k units/day, IP) was injected for seven consecutive days to prevent infection. The rats were housed individually after the surgery and allowed 3–5 days of recovery before behavioral experiments.

### Effect of glial cell-derived neurotrophic factor on morphine-induced conditioned place preference in rats

Forty-eight CPP trained rats were randomly divided into six groups (*n* = 8), namely IV-NS group [intravenous (IV), normal saline (NS)], IV-GDNF group (IV, GDNF 3 mg/kg), M group (blank microbubbles, IV), M-GDNF group (GDNF microbubbles, IV), IN-NS group [intranuclear (IN) NS injected into VTA] and IN-GDNF group (IN-GDNF 10 μg/μL injected into VTA). The volume of administration was 0.5 ml in all IV injection groups and 0.5 μL in the IN-injection groups. After drug administration, MRI-guided focused ultrasound was performed in the M and M-GDNF groups. 0.5 ml NS was administered to an additional group of rats during the entire study period as control (Con group). The method used in this study for preparing the GDNF-loaded MBs followed the protocol described in our previously published papers (Lin et al., 2015; Lin et al., 2016). The above treatments were performed on the 2nd and 4th day after completion of the CPP test, and an additional CPP test was performed on the 5th day to observe the changes in each group. The rats were decapitated immediately after the last measurement, and the brain tissue of half the rats in each group was used for Western blot analysis, while the other half was analyzed with HPLC (High-performance liquid chromatography).

### Focused ultrasound program

The focused ultrasound system was placed in a 3.0 T MRI chamber for brain tissue imaging and ultrasound localization. The T1-phase scan was used to detect brain tissue in real time and locate brain regions. The right hemisphere of the brain was irradiated by focused ultrasound (frequency, 1 MHz; MB dosage, 0.5 ml; exposure time, 1 min; pressure amplitude, 0.8 MPa; delay time, 60 s). After ultrasound irradiation and MRI image acquisition, Evans blue (EB, 100 mg/kg) was injected through the tail vein to reveal the BBB open area. Rats that did not receive ultrasound were used as controls.

### Effects of glial cell-derived neurotrophic factor on withdrawal symptoms

A morphine withdrawal model was established using IP naloxone (Sigma, United States) (4 mg/kg) 4 h after the last injection of morphine in addiction rats to induce withdrawal symptoms. The grouping and administration methods of rats were the same as described above. The treatments were performed 24 and 72 h before naloxone-induced withdrawal. The withdrawal symptoms were observed and scored based on wet dog shaking, abnormal posture, irritability, clenching, vegetative nervous system symptoms, and weight loss. The rats were decapitated immediately after the test, and the brain tissues were analyzed by Western blot and HPLC as described above.

### Western blot

The olfactory bulb and cerebellum of rats’ brains were removed, and the telencephalon was reserved, placed in n-hexane at −60°C for 20 s, and then placed in a pre-cooled (−20°C) frozen sectioning machine. The brain tissues were embedded with tissue-embedding agent (OCT) and fixed. Continuous 50-μm thickness sectioning was performed until the VTA area was exposed according to the stereotaxic map of the rat brain. Then, the VTA was removed using a syringe needle (size 16) and placed into a cooled Eppendorf tube. RIPA cracking solution containing 1 mM phenylmethyl sulfonyl fluoride (PMSF) was added (150–200 μL/20 mg) and homogenized mechanically three times (15 s each with 5 s apart). After full lysis, centrifugation at 12,000 rpm/min for 5 min was performed, supernatant taken, protein content determined using the BCA method, and sample concentrations in each group adjusted to the same level using lysis solution. All of the operations above were performed on the ice. The 4X protein loading buffer was proportionally added, and the protein was heated in boiling water (3 min) for albuminous degeneration and frozen at −70°C for later use.

A gel caster was successively filled with 10% separated glue and 5% concentrated glue. Marker (2 μL/well) and protein samples (20 μL/well) were successively added, and sodium dodecyl sulfate-polyacrylamide gel electrophoresis (SDS-PAGE) was performed with a constant voltage of 80 V for concentrated glue and 120 V for separated glue. At the end of the electrophoresis, the gel was removed and electro-transferred using a polyvinylidene difluoride (PVDF) at a constant current of 250 mA for 2.5 h. Then, the PVDF membrane was intruded into 5% bovine serum albumin (BSA) and sealed for 2 h. Subsequently, 5% BSA-diluted primary antibody (1:1,000) was added and incubated overnight in the refrigerator at 4°C, and then rinsed three times for 5 min each using TBST. TBST-diluted secondary antibody (1:2,000) was added and incubated at room temperature for 45 min, then rinsed with TBST three times for 5 min each. ECL chemiluminescence solution was used for development to compare the expression level of tyrosine hydroxylase (TH) (Abcam, United Kingdom) in the VTA region of rats in each group.

### high performance liquid chromatography test

The rat brain samples were thawed at room temperature, and 500 μL of perchloric acid (0.4 M) were added, mixed, and centrifuged at 15,000 rpm for 3 min. Supernatant (100 μL) was added to 200 μL mobile phase, and 20 μL of the mixture were injected into the chromatography. The peak area was recorded, and was quantified using the standard curve. The standard solution series were determined, and the peak area and concentration were calculated using least square linear regression. The chromatographic conditions were as follows: column, diamond-C18 (250 mm × 4.6 mm, 5 μm); mobile phase, 4.04 g anhydrous sodium acetate, 0.80 g heptane sodium sulfonate, and 0.188 g EDTA dissolved in 1,000 ml water, filtered using 0.25 μm filter membrane, pH adjusted to 4.5 with acetic acid, then to 3.8 with phosphoric acid, and 220 ml methanol added; flow rate, 1.0 ml/min; detector potential, 0.75 V; column temperature, 35°C; and injection volume: 20 μL. Dopamine (DA) hydrochloride (Sigma, United States), norepinephrine (NE) (Sigma, United States), and 5-hydroxytryptamine (5-HT) hydrochloride (Sigma, United States) were dissolved in methanol to prepare a standard solution for control. The levels of NE, DA, and 5-HT of NAc were determined.

### Statistical analysis

The Western blot strips were analyzed with Quantity One software. The corresponding background box was cut out with the value of the strip box, and the value of the strip was obtained. According to the same sample, the value of each experimental group was compared with the corresponding blank control group, and the ratio was obtained. The measurement data results were expressed as mean ± SEM and analyzed by SPSS 26.0. One-way ANOVA was used for comparison between groups, and *p* < 0.05 was considered statistically significant.

## Results

### Preparation and characterization of glial cell-derived neurotrophic factor-loaded microbubbles

**FIGURE 1:**
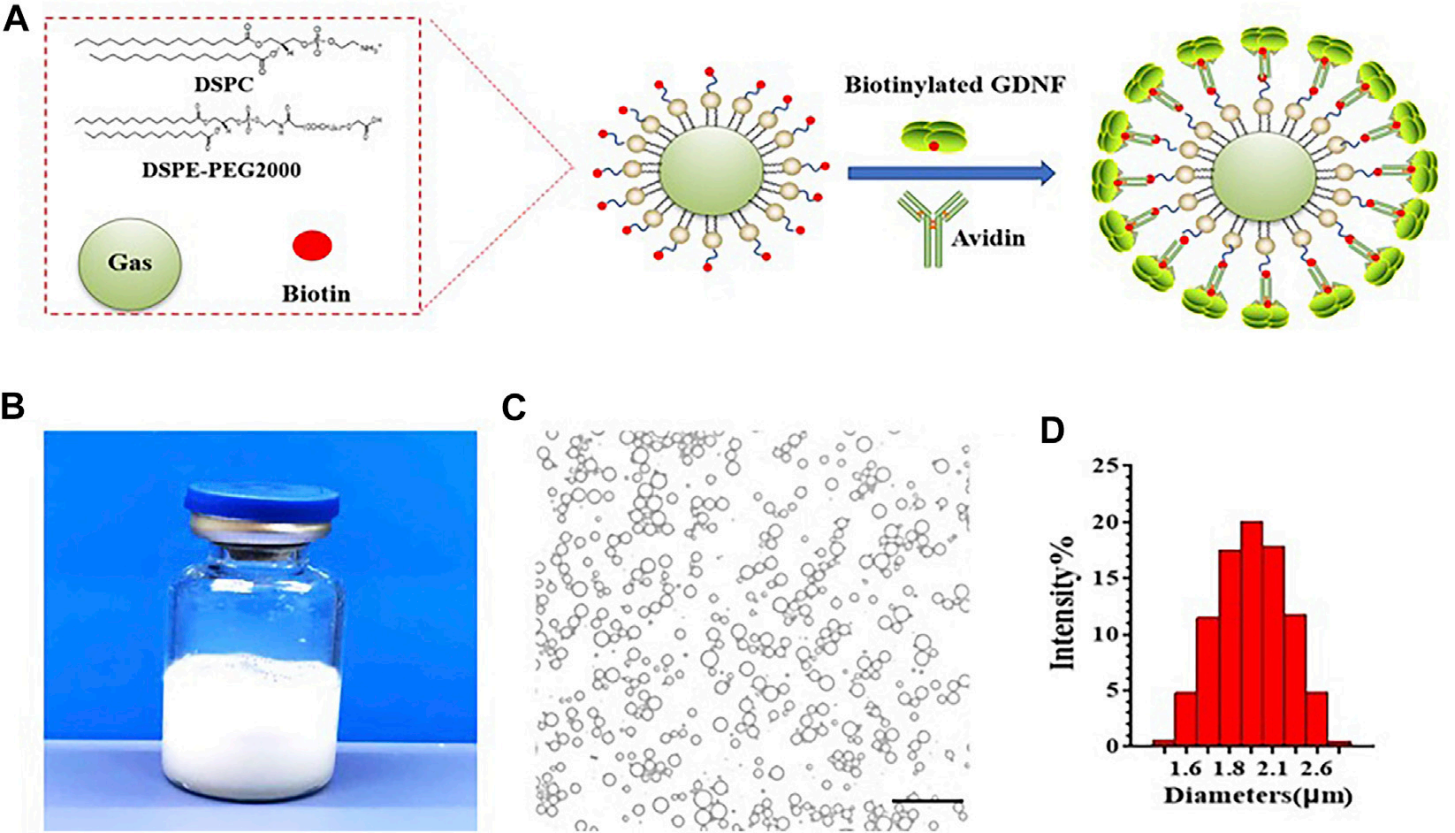
GDNF-loaded MBs. **(A)** Diagram of GDNF-loaded MBs. **(B)** Appearance of GDNF-loaded MBs. **(C)** Microscopic examination of GDNF-loaded MBs. **(D)** Particle size detection of GDNF-loaded MBs. GDNF, glial cell-derived neurotrophic factor; MBs, microbubbles. Scale bar = 20 µm.

GDNF-loaded MBs were successfully prepared. A schematic illustration of the structure of M-GDNF is presented in Figure 1A. Figure 1B showed the appearance of the prepared GDNF-loaded MBs in vial. The morphologic appearance of MB-GDNF after conjugating with GDNF was observed through bright-field microscopy (Figure 1C). The mean diameter and concentration of the GDNF-loaded MBs were 1.8 ± 0.5 µm and (2.15 ± 0.4) × 10^10^ MB/ml, respectively (Figure 1D).

### Morphine-induced conditioned place preference in rats

**FIGURE 2:**
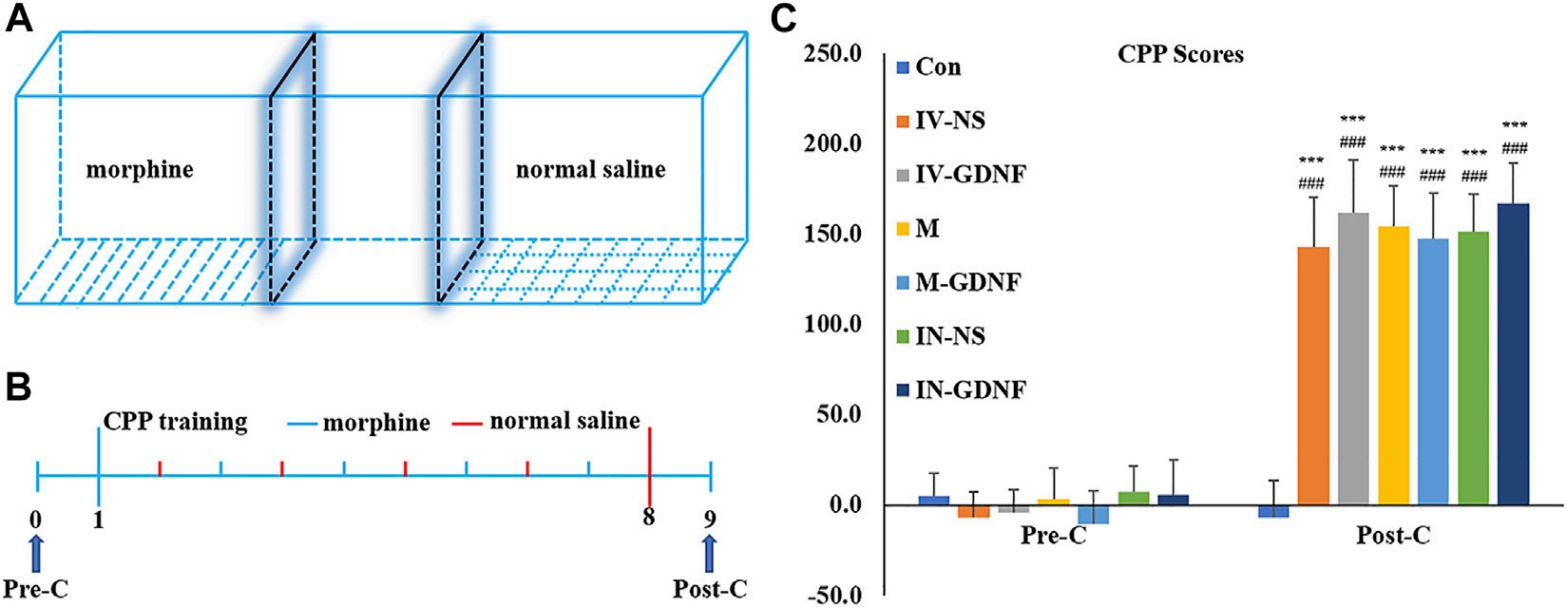
Morphine-induced CPP in rats. **(A)** CPP training diagram. **(B)** CPP training protocol. **(C)** CPP scores. Means ± SEM, *n* = 8, ***p < 0.001 vs*.* Pre-C; ^###^ *p* < 0.001 vs*.* control group. CPP, conditioned place preference.

Repeated measures ANOVA was used to analyze behavioral data. The results showed that after morphine CPP training, all rats acquired significant CPP, and CPP scores were significantly higher in the CPP training groups (post-C) compared with pre-C (*p* < 0.001). CPP scores in CPP training groups were significantly higher than those in the control group (*p* < 0.001). There was no significant difference in the control group before and after training (*p* > 0.05) (Figure 2).

### Effect of focused ultrasound combined with glial cell-derived neurotrophic factor-loaded microbubbles on morphine-induced conditioned place preference in rats

**FIGURE 3:**
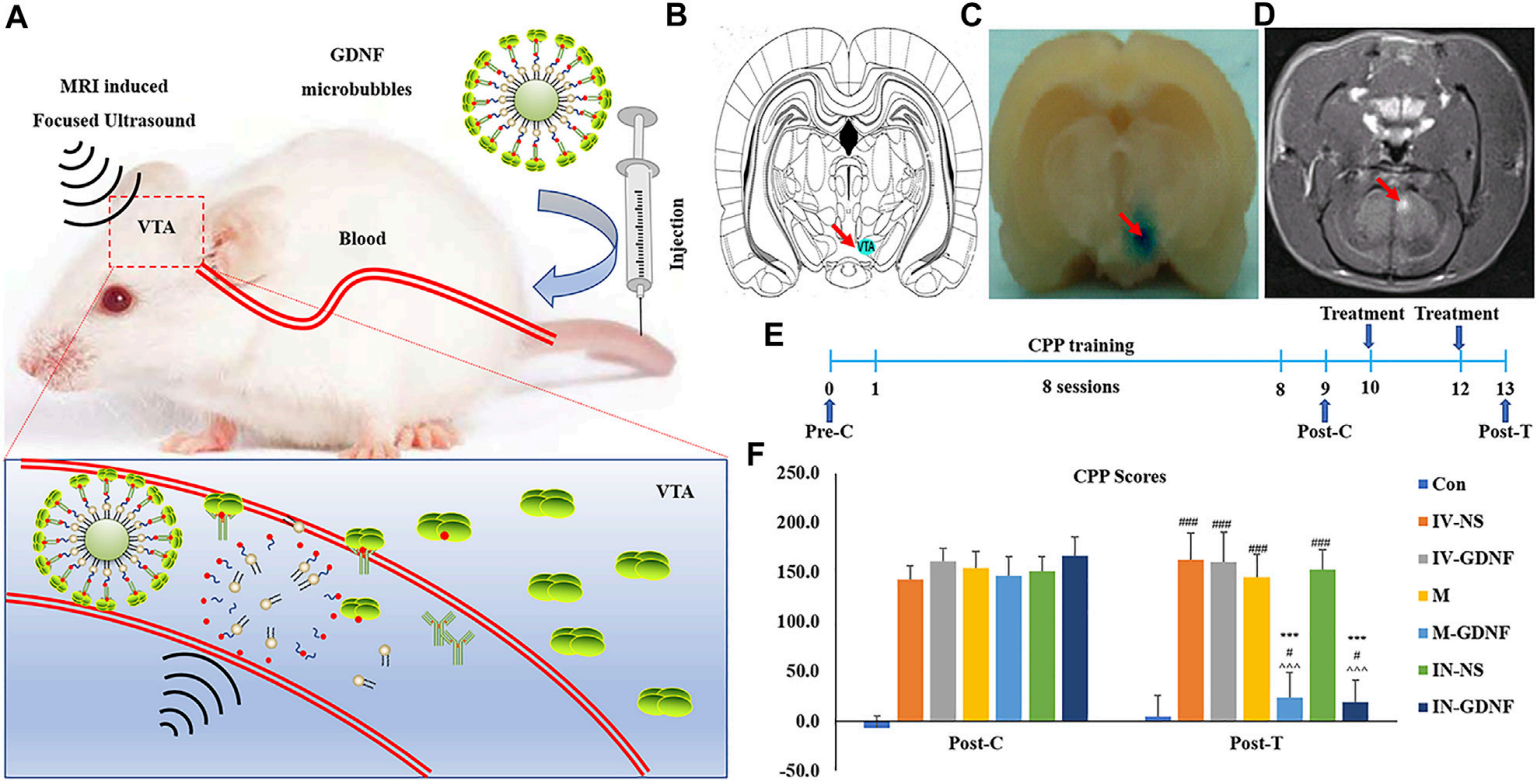
Focused ultrasound combined with GDNF-loaded MBs reversed morphine-induced CPP in rats. **(A)** Schematic diagram of targeted delivery of GDNF to VTA via focused ultrasound combined with MBs. **(B)** Schematic representations of VTA. **(C)** The location of the BBB opening was confirmed by EB staining of the affected area (arrows). **(D)** BBB opening was monitored by leakage of the MR contrast agent into the brain parenchyma on coronal (COR) MR images (arrows). **(E)** Schematic diagram of experimental process. **(F)** CPP scores. Means ± SEM, *n* = 8, ***p < 0.001 vs*.* Post-C; ^#^ *p* < 0.05, ^###^ *p* < 0.001 vs*.* Control group; ^^^*p* < 0.001 vs*.* IV-NS group. BBB, blood-brain barrier; CPP, conditioned place preference; MBs, microbubbles; MR, magnetic resonance; VTA, ventral tegmental area.

The principle and method of GDNF-targeted delivery to the VTA by focused ultrasound combined with MBs are shown in Figure 3A. The MRI-guided focused ultrasound irradiation to the VTA can partially open the BBB in this region. The schematic representations showed the location of VTA (Figure 3B). As shown in Figure 3C, EB staining proved that MRI-guided focused ultrasound successfully opened the BBB in the VTA region using the previously measured optimal parameter conditions, which was further confirmed by MRI imaging (Figure 3D). The CPP training and treatment procedures for rats are shown in Figure 3E. CPP test results showed that the test time had a significant effect on morphine-induced CPP scores [F (2,98) = 719.07, *p* < 0.01], and there was also a significant interaction between each treatment group and the test time [F (12,98) = 54.63, *p* < 0.01]. Post hoc analysis showed that the M-GDNF and IN-GDNF CPP scores were significantly lower than those of the IV-NS group and that test before treatment (post-C), and showed no significant differences compared with the control group. Furthermore, there was no significant difference between the M-GDNF and IV-GDNF groups (Figure 3F). The morphine-induced CPP was not significantly affected by GDNF injection (IV-GDNF) or MBs (M) via caudal vein or NS injected intranuclearly (IN-NS). These results confirm that GDNF can reverse morphine-induced CPP, and targeted delivery of GDNF via focused ultrasound combined with MBs can achieve therapeutic efficacy comparable to that of direct intranuclear injection.

### Influences of focused ultrasound combined with glial cell-derived neurotrophic factor-loaded microbubbles on neurotransmitters in addiction rats

**FIGURE 4:**
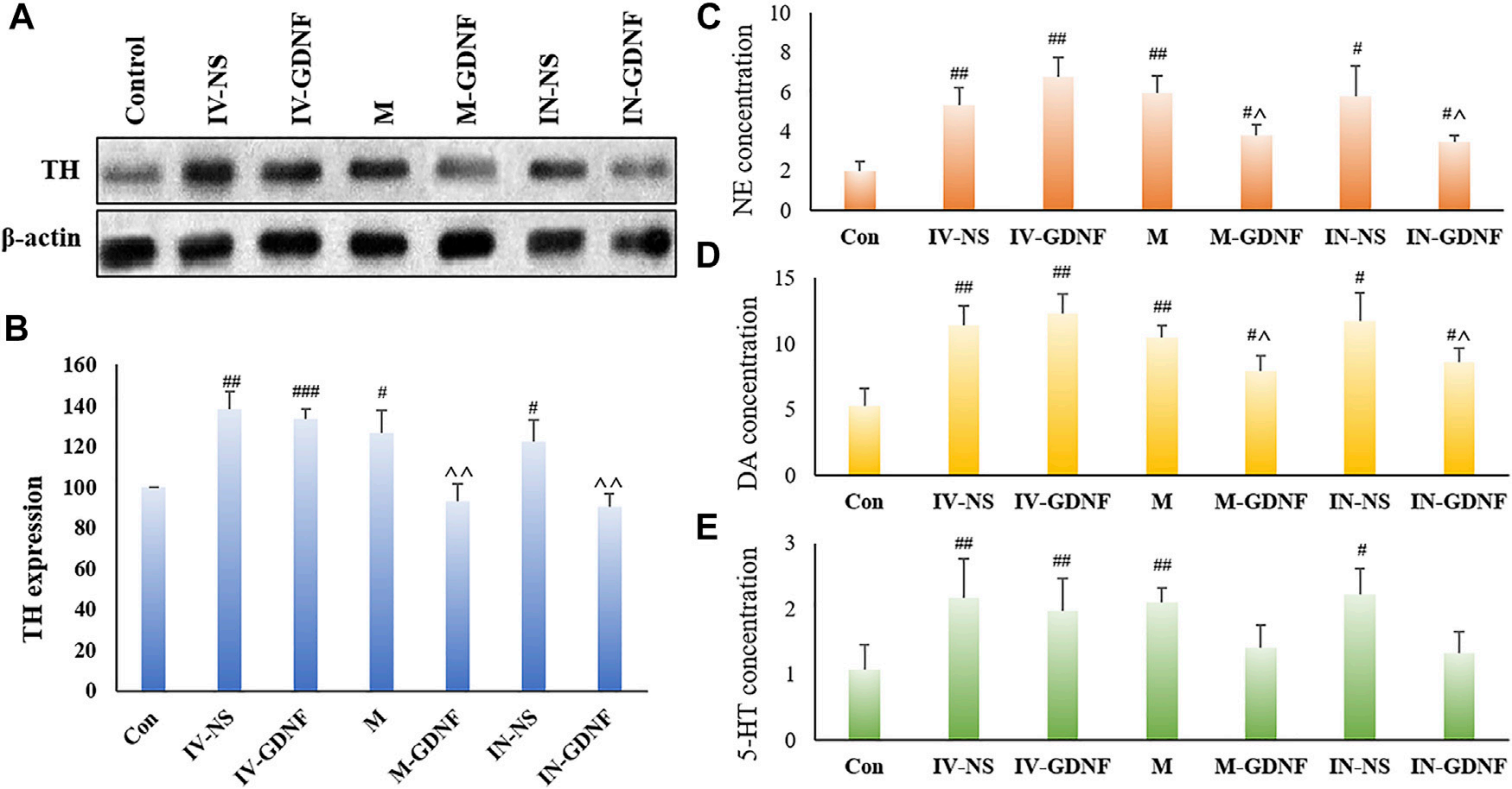
Effect of different treatments on morphine neurotransmitters of addiction rats. **(A)** Representative bands of TH expression. **(B)** Analysis of TH expression in different groups. **(C)**. NE concentration measured by HPLC. **(D)** DA concentration measured by HPLC. **(E)**. 5-HT concentration measured by HPLC. Means ± SEM, *n* = 3, ^#^ *p* < 0.05, ^##^ *p* < 0.01 vs*.* control group; ^^^ *p* < 0.05 vs*.* IV-SN group. DA, dopamine; HPLC, high performance liquid chromatography; 5-HT, serotonin; NE, norepinephrine; TH, tyrosine hydroxylase.

One-way ANOVA was used for analysis. Western blot results showed that morphine significantly up-regulated TH expression in the VTA region of rats (Figures 4A,B), TH expression levels of M-GDNF and IN-GDNF groups was significantly down-regulated compared with the IV-NS group, and there was no significant difference between the M-GDNF, IN-GDNF, and control groups (Figure 4B). The contents of NE, DA, and 5-HT in the NAc region were detected by HPLC. The results showed that morphine could significantly induce an increase of NE, DA, and 5-HT in the NAc region of rats, while M-GDNF and IN-GDNF treatment could significantly reverse the increase of NE and DA induced by morphine (Figures 4C,D). For 5-HT, the levels of 5-HT in the M-GDNF and IN-GDNF groups were not significantly different from those in the IV-NS and control groups, suggesting that M-GDNF and IN-GDNF treatments could partially reverse the increase of 5-HT induced by morphine (Figure 4E).

### Effect of focused ultrasound combined with glial cell-derived neurotrophic factor-loaded microbubbles on morphine withdrawal symptoms in rats

Naloxone (4 mg/kg, IP) administration 4 h after the last injection of morphine induced obvious withdrawal symptoms in rats. Rats in different groups received corresponding treatments 24 h before naloxone injection to observe the effects on morphine withdrawal symptoms. The results are shown in Table 1. Among the ten withdrawal symptoms observed, the M-GDNF group had five symptoms (jumping, wet dog shakes, exploring, diarrhea, and weight loss) and the IN-GDNF group had six symptoms (jumping, wet dog shakes, exploring, piloerection, diarrhea, and weight loss) scored significantly lower than those in other treatment groups (*p* < 0.05). Compared with the IN-GDNF group, only the scores of wet dog shakes were significantly higher in the M-GDNF group. These results confirm that GDNF can significantly reduce the withdrawal symptoms of morphine, and focused ultrasound combined with MBs-targeted delivery of GDNF can achieve similar therapeutic effects comparable to those of intranuclear injection.

**TABLE 1:** Effect of different treatments on naloxone-induced morphine withdrawal symptoms.

Withdrawal sign	Control	IV-NS	IV-GDNF	M	M-GDNF	IN-NS	IN-GDNF
Jumping	0.63 ± 0.52	2.88 ± 0.99^***^^^$^	2.75 ± 0.71^***^^$^	3.25 ± 1.28^***^^^^$$^	1.75 ± 0.71^*^^#^	4.13 ± 1.64^***^^^^^$$$^	1.50 ± 0.93
Teeth Chattering	0.00 ± 0.00	2.13 ± 0.99^***^	2.50 ± 0.93^***^^$^	2.00 ± 0.76^***^	1.75 ± 0.89^***^	1.88 ± 0.83^***^	1.63 ± 0.74^***^
Writhing	0.00 ± 0.00	2.38 ± 0.92^***^	2.75 ± 0.89^***^	3.13 ± 0.83^***^^^$^	2.25 ± 1.04^***^	3.00 ± 1.07^***^^$^	2.00 ± 0.76^***^
Wet-dog Shakes	0.00 ± 0.00	7.00 ± 1.60^***^^^^^$$$^	6.63 ± 1.06^***^^^^$$$^	6.13 ± 1.81^***^^^$$$^	4.38 ± 1.41^***^	7.25 ± 1.28^***^^^^^$$$^	2.88 ± 1.25^***^^^^
Exploring	1.13 ± 0.64	8.50 ± 1.85^***^^^^$$$^	9.00 ± 1.31^***^^^^^$$$^	9.75 ± 2.25^***^^^^^$$$^	5.63 ± 1.19^***^	10.38 ± 2.07^***^^^^^$$$^	4.63 ± 1.69^***^
Ptosis	0.00 ± 0.00	6.00 ± 1.77^***^^^$$^	5.00 ± 1.31^***^	5.38 ± 1.41^***^	4.38 ± 1.19^***^	5.88 ± 1.46^***^^^$$^	4.13 ± 1.13^***^
Piloerection	0.00 ± 0.00	3.00 ± 1.41^***^^$$^	2.88 ± 1.25^***^^$^	3.25 ± 1.04^***^^$$^	2.63 ± 0.92^***^	3.63 ± 1.19^***^^$$$^	1.50 ± 0.76^**^^^^
Irritability	0.00 ± 0.00	1.13 ± 0.64^***^	1.38 ± 0.74^***^	1.50 ± 0.53^***^^$^	1.00 ± 0.53^***^	1.63 ± 0.74^***^^^$^	0.88 ± 0.35^***^
Diarrhea	0.00 ± 0.00	4.25 ± 1.04^***^^^^$$$^	5.38 ± 1.06^***^^^^^$$$^	4.50 ± 1.20^***^^^^^$$$^	2.50 ± 0.93^***^	5.13 ± 1.25^***^^^^^$$$^	2.25 ± 0.89^***^
Weight Loss	1.48 ± 0.49	5.18 ± 1.45^***^^^^$$^	4.90 ± 1.21^***^^^$$^	6.05 ± 1.44^***^^^^^$$$^	3.35 ± 1.28^**^	6.54 ± 1.61^***^^^^^$$$^	2.88 ± 1.11^*^

Mean ± SEM. *p < 0.05, **p < 0.01, ***p < 0.001 vs*.* control group; ^^^ *p* < 0.05, ^^^^ *p* < 0.01, ^^^^^ *p* < 0.001 vs*.* M-GDNF, group; ^$^ *p* < 0.05, ^$$^ *p* < 0.01, ^$$$^ *p* < 0.001 vs*.* IN-GDNF, group.

### Possible mechanisms of different treatments affecting morphine withdrawal symptoms

**FIGURE 5:**
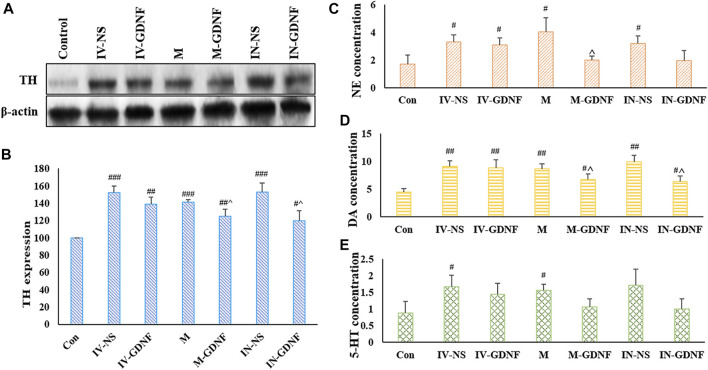
Effect of different treatments on neurotransmitters of morphine withdrawal rats. **(A)** Representative bands of TH expression. **(B)** Analysis of TH expression in different groups. **(C)** NE concentration measured by HPLC. **(D)** DA concentration measured by HPLC. **(E)** 5-HT concentration measured by HPLC. Means ± SEM, *n* = 3, ^#^ *p* < 0.05, ^##^ *p* < 0.01, ^###^ *p* < 0.001 vs*.* Control group; ^^^ *p* < 0.05 vs*.* IV-SN group. DA, dopamine; HPLC, high performance liquid chromatography; 5-HT, serotonin; NE, norepinephrine; TH, tyrosine hydroxylase.

The western blot results showed that TH expression in the VTA of morphine withdrawal rats was significantly higher than that of the control group. TH expression levels in the IN-GDNF and M-GDNF groups were significantly lower than in the IV-NS group, and there was no significant difference between the M-GDNF and IN-GDNF groups (Figures 5A,B). The contents of NE, DA and 5-HT in the NAc region were determined by HPLC. The results showed that morphine withdrawal significantly increased the NE, DA, and 5-HT contents in the NAc region of rats, and the up-regulation of NE and DA were significantly reversed by M-GDNF and IN-GDNF treatment (Figures 5C,D). The levels of 5-HT in the M-GDNF and IN-GDNF groups showed no significant difference compared with both the IV-NS and control group, suggesting that M-GDNF and IN-GDNF treatments can partially reverse the increase of 5-HT induced by morphine withdrawal (Figure 5E).

## Discussion

The abuse of addictive substances has become a crucial factor affecting world economic development, endangering public health, and threatening social harmony and stability (Maier et al., 2020). Drug abuse can not only cause direct economic losses and increase the probability of crime, but also cause the rapid spread of HIV, HBV, and other infectious diseases (Messer et al., 2000). Therefore, it is of great significance to seek treatment drugs for addiction. The BBB is a vital structure that maintains the stability of the central nervous system. However, the actions of many drugs for addiction treatment, especially macromolecules, are limited due to their inability to cross the BBB and enter the brain. This is a complex problem in the development of drugs for addiction treatment (Nelson, 2006). In recent years, focused ultrasound has been regarded as an effective non-invasive method to open the BBB and achieve targeted drug delivery (United Nation, 2021). This technology may provide new approaches and strategies for the treatment of addiction.

As one of the most representative members of the neurotrophic factor family, GDNF is a large protein with a molecular weight of 24 kDa. GDNF is widely present in the developing central nervous system and mature brain tissue, and can promote the growth, protection, and repair of various neurons, such as glial cells and serotonergic and dopaminergic neurons (Sajadi et al., 2005). GDNF receptors are expressed in many brain regions, including the cerebellum, hypothalamic nucleus, amygdala, and hippocampus, but are mainly found in the SNc and VTA. Furthermore, dopaminergic neurons in the VTA region are closely associated with addiction (Shi et al., 2018). TH is a rate-limiting enzyme of DA synthesis, and the up-regulation of TH levels is considered one of the biomarkers of addiction. Direct injection of GDNF into the VTA region reversed CPP, NR1 subunit of the NMDA receptor, and TH expression in the brain of cocaine-trained rats (Sun et al., 2018). Cocaine increases extracellular DA concentration by blocking synaptic level reuptake, whereas morphine increases DA concentration by stimulating DA neurons in the VTA region (Tosi et al., 2020). It was also found that GDNF could regulate TH activity, and the increase in GDNF levels was closely related to the decrease of TH activity and DA levels in the striatum (Uhl et al., 2019). In addition, injection of GDNF in the VTA region can reverse the elevation of TH protein induced by cocaine and morphine (Sun et al., 2018; Volkow and Boyle, 2018). These studies indicate that GDNF can attenuate the biochemical and behavioral changes of drug abuse, is an effective substance for addiction treatment (Sun et al., 2018; Volkow et al., 2019), and its mechanism may be related to the regulation of TH activity.

Although GDNF has shown potential in the treatment of drug addiction, its difficulty in penetrating the BBB severely limits its use (Wang et al., 2012; Wang et al., 2020). Therefore, a fundamental scientific problem in using GDNF for the treatment of drug addiction is how to promote passage of GDNF through the BBB to achieve effective concentrations in the central nervous system. In this study, we used focused ultrasound combined with GDNF MBs to achieve targeted delivery into the VTA brain region. Our previous study proved that the optimal parameters for ultrasound combined MB opening of the BBB are as follows: 1 MHz frequency, 0.5 ml MB dose, 1 min irradiation time, 0.8 MPa sound pressure, and 60 s delay time (Lin et al., 2015; Lin et al., 2016). These were the parameters used in this study for focused ultrasound-targeted irradiation of the VTA under MRI guidance to open the BBB locally. EB and MRI contrast agent leakage in the local open area were consistent with the VTA positions shown in the model figure, indicating that our study could accurately open the BBB locally and provide a basis for the targeted release of GDNF.

To verify the inhibitory effect of an increased content of GDNF in the brain, achieved through ultrasound combined with GDNF MB opening of the BBB, on morphine addiction, morphine CPP and morphine withdrawal rat models were established. After morphine training, all rats except the control group acquired significant CPP. Compared with the control group, there were significant differences between the M-GDNF and IN-GDNF groups and other groups after the corresponding treatments were given to each. Naloxone can induce significant morphine withdrawal symptoms. In the M-GDNF group, five of ten withdrawal symptoms scores were significantly lower than those of the other treatment groups, while there were no significant differences with the IN-GDNF group except for wet dog shakes. The results showed that ultrasound combined with GDNF MB to open the BBB and increase the content of central GDNF could destroy the CPP and relieve the withdrawal symptoms of morphine addiction in rats and achieve the same effect as brain microinjection of GDNF.

Furthermore, we investigated the effects of increasing the brain content of GDNF via ultrasound combined with GDNF MB on the BBB opening on TH expression and content of mono-ammonia neurotransmitters such as NE, DA, and 5-HT in order to reveal the mechanism of action. The results showed that in both the morphine-induced CPP and naloxone-induced withdrawal models, ultrasound combined with GDNF MB group (M-GDNF) could significantly reverse the increased TH expression and content of NE, DA, and 5-HT, and achieved similar effects to direct injections of GDNF into the brain VTA (IN-GDNF).

## Conclusion

This study provides a non-invasive and reversible method to open the BBB, target delivery of GDNF to the VTA region, and treat morphine addiction. This technique can avoid brain tissue damage caused by intracranial microinjection, and further increases the advantages of neurotrophic factors, such as GDNF, in the treatment of brain diseases. It provides a new and effective way to explore the application of peripheral macromolecular drugs in the treatment of neuropsychiatric diseases such as addiction.

## Data Availability

The original contributions presented in the study are included in the article/supplementary materials, further inquiries can be directed to the corresponding authors.

## References

[B1] Allen S. J. Watson J. J. Shoemark D. K. Barua N. U. Patel N. K. (2013). GDNF, NGF and BDNF as Therapeutic Options for Neurodegeneration. Pharmacol. Ther. 138 (2), 155–175. 10.1016/j.pharmthera.2013.01.004 23348013

[B2] Becker W. C. Fiellin D. A. (2020). When Epidemics Collide: Coronavirus Disease 2019 (COVID-19) and the Opioid Crisis. Ann. Intern. Med. 173 (1), 59–60. 10.7326/m20-1210 32240291 PMC7138333

[B3] Belin D. Deroche-Gamonet V. Jaber M. (2007). Cocaine-induced Sensitization Is Associated with Altered Dynamics of Transcriptional Responses of the Dopamine Transporter, Tyrosine Hydroxylase, and Dopamine D2 Receptors in C57Bl/6J Mice. Psychopharmacol. Berl. 193 (4), 567–578. 10.1007/s00213-007-0790-3 17505818

[B4] Bleier B. S. Kohman R. E. Guerra K. Nocera A. L. Ramanlal S. Kocharyan A. H. (2016). Heterotopic Mucosal Grafting Enables the Delivery of Therapeutic Neuropeptides across the Blood Brain Barrier. Neurosurgery 78 (3), 448–457. 10.1227/neu.0000000000001016 26352099

[B5] Carnicella S. Kharazia V. Jeanblanc J. Janak P. H. Ron D. (2008). GDNF Is a Fast-Acting Potent Inhibitor of Alcohol Consumption and Relapse. Proc. Natl. Acad. Sci. U. S. A. 105 (23), 8114–8119. 10.1073/pnas.0711755105 18541917 PMC2423415

[B6] Carnicella S. Ron D. (2009). GDNF--a Potential Target to Treat Addiction. Pharmacol. Ther. 122 (1), 9–18. 10.1016/j.pharmthera.2008.12.001 19136027 PMC3682485

[B7] Chen W. Hu Y. Ju D. (2020). Gene Therapy for Neurodegenerative Disorders: Advances, Insights and Prospects. Acta Pharm. Sin. B 10 (8), 1347–1359. 10.1016/j.apsb.2020.01.015 32963936 PMC7488363

[B8] Cheron J. Kerchove d'Exaerde A. (2021). Drug Addiction: from Bench to Bedside. Transl. Psychiatry 11 (1), 424. 10.1038/s41398-021-01542-0 34385417 PMC8361217

[B9] Cintrón-Colón A. F. Almeida-Alves G. Boynton A. M. Spitsbergen J. M. (2020). GDNF Synthesis, Signaling, and Retrograde Transport in Motor Neurons. Cell. Tissue Res. 382 (1), 47–56. 10.1007/s00441-020-03287-6 32897420 PMC7529617

[B10] Everitt B. J. Robbins T. W. (2016). Drug Addiction: Updating Actions to Habits to Compulsions Ten Years on. Annu. Rev. Psychol. 67, 23–50. 10.1146/annurev-psych-122414-033457 26253543

[B11] Fu A. Zhou Q. H. Hui E. K. Lu J. Z. Boado R. J. Pardridge W. M. (2010). Intravenous Treatment of Experimental Parkinson's Disease in the Mouse with an IgG-GDNF Fusion Protein that Penetrates the Blood-Brain Barrier. Brain Res. 1352, 208–213. 10.1016/j.brainres.2010.06.059 20599807 PMC2926206

[B12] Gasca-Salas C. Fernández-Rodríguez B. Pineda-Pardo J. A. Rodríguez-Rojas R. Obeso I. Hernández-Fernández F. (2021). Blood-brain Barrier Opening with Focused Ultrasound in Parkinson's Disease Dementia. Nat. Commun. 12 (1), 779. 10.1038/s41467-021-21022-9 33536430 PMC7859400

[B13] Ghitza U. E. Zhai H. Wu P. Airavaara M. Shaham Y. Lu L. (2010). Role of BDNF and GDNF in Drug Reward and Relapse: a Review. Neurosci. Biobehav. Rev. 35 (2), 157–171. 10.1016/j.neubiorev.2009.11.009 19914287 PMC2891859

[B14] Holt E. (2021). Drug Legislation May Be Key to Ending HIV Epidemic. Lancet HIV 8 (7), e392–e393. 10.1016/s2352-3018(21)00131-4 34197770

[B15] Kastin A. J. Akerstrom V. Pan W. (2003). Glial Cell Line-Derived Neurotrophic Factor Does Not Enter Normal Mouse Brain. Neurosci. Lett. 340 (3), 239–241. 10.1016/s0304-3940(03)00007-7 12672550

[B16] Kilic U. Kilic E. Dietz G. P. Bähr M. (2003). Intravenous TAT-GDNF Is Protective after Focal Cerebral Ischemia in Mice. Stroke 34 (5), 1304–1310. 10.1161/01.Str.0000066869.45310.50 12677018

[B17] Lin C. Y. Hsieh H. Y. Chen C. M. Wu S. R. Tsai C. H. Huang C. Y. (2016). Non-invasive, Neuron-specific Gene Therapy by Focused Ultrasound-Induced Blood-Brain Barrier Opening in Parkinson's Disease Mouse Model. J. Control. Release 235, 72–81. 10.1016/j.jconrel.2016.05.052 27235980

[B18] Lin C. Y. Hsieh H. Y. Pitt W. G. Huang C. Y. Tseng I. C. Yeh C. K. (2015). Focused Ultrasound-Induced Blood-Brain Barrier Opening for Non-viral, Non-invasive, and Targeted Gene Delivery. J. Control. Release 212, 1–9. 10.1016/j.jconrel.2015.06.010 26071631

[B19] Lin C. Y. Lin Y. C. Huang C. Y. Wu S. R. Chen C. M. Liu H. L. (2020). Ultrasound-responsive Neurotrophic Factor-Loaded Microbubble- Liposome Complex: Preclinical Investigation for Parkinson's Disease Treatment. J. Control. Release 321, 519–528. 10.1016/j.jconrel.2020.02.044 32112852

[B20] Lin C. Y. Tsai C. H. Feng L. Y. Chai W. Y. Lin C. J. Huang C. Y. (2019). Focused Ultrasound-Induced Blood Brain-Barrier Opening Enhanced Vascular Permeability for GDNF Delivery in Huntington's Disease Mouse Model. Brain Stimul. 12 (5), 1143–1150. 10.1016/j.brs.2019.04.011 31079989

[B21] Maier H. B. Neyazi M. Neyazi A. Hillemacher T. Pathak H. Rhein M. (2020). Alcohol Consumption Alters Gdnf Promoter Methylation and Expression in Rats. J. Psychiatr. Res. 121, 1–9. 10.1016/j.jpsychires.2019.10.020 31710958

[B22] Messer C. J. Eisch A. J. Carlezon W. A. Jr. Whisler K. Shen L. Wolf D. H. (2000). Role for GDNF in Biochemical and Behavioral Adaptations to Drugs of Abuse. Neuron 26 (1), 247–257. 10.1016/s0896-6273(00)81154-x 10798408 PMC4451194

[B23] Nelson R. (2006). Blood-brain Barrier Challenged by New Drug Delivery Vehicle. Lancet Neurol. 5 (2), 117. 10.1016/s1474-4422(06)70340-4 16456936

[B24] Sajadi A. Bauer M. Thöny B. Aebischer P. (2005). Long-term Glial Cell Line-Derived Neurotrophic Factor Overexpression in the Intact Nigrostriatal System in Rats Leads to a Decrease of Dopamine and Increase of Tetrahydrobiopterin Production. J. Neurochem. 93 (6), 1482–1486. 10.1111/j.1471-4159.2005.03139.x 15935064

[B25] Shi Y. Wang F. Hu A.-Z. Wang Q.-W. Wu J.-L. Li M.-H. (2018). Effects and Mechanisms of Jinniu Capsule on Methamphetamine-Induced Conditioned Place Preference in Rats. Open Chem. 16 (1), 674–680. 10.1515/chem-2018-0074

[B26] Sun Y. Zhang Y. Zhang D. Chang S. Jing R. Yue W. (2018). GABRA2 Rs279858-Linked Variants Are Associated with Disrupted Structural Connectome of Reward Circuits in Heroin Abusers. Transl. Psychiatry 8 (1), 138. 10.1038/s41398-018-0180-0 30061709 PMC6066482

[B27] Tosi G. Duskey J. T. Kreuter J. (2020). Nanoparticles as Carriers for Drug Delivery of Macromolecules across the Blood-Brain Barrier. Expert Opin. Drug Deliv. 17 (1), 23–32. 10.1080/17425247.2020.1698544 31774000

[B28] Uhl G. R. Koob G. F. Cable J. (2019). The Neurobiology of Addiction. Ann. N. Y. Acad. Sci. 1451 (1), 5–28. 10.1111/nyas.13989 30644552 PMC6767400

[B29] United Nation (2021). World Drug Report 2021.

[B30] Volkow N. D. Boyle M. (2018). Neuroscience of Addiction: Relevance to Prevention and Treatment. Am. J. Psychiatry 175 (8), 729–740. 10.1176/appi.ajp.2018.17101174 29690790

[B31] Volkow N. D. Jones E. B. Einstein E. B. Wargo E. M. (2019). Prevention and Treatment of Opioid Misuse and Addiction: A Review. JAMA Psychiatry 76 (2), 208–216. 10.1001/jamapsychiatry.2018.3126 30516809

[B32] Wang F. Li N. Wei X. Jia X. Liu H. Wang Y. (2020). MRI-guided Focused Ultrasound-Induced Blood Brain Barrier Disruption to Deliver Glial Cell Line Derived Neurotropic Factor Proteins into Brain to Treat Rat Depression. J. Biomed. Nanotechnol. 16 (5), 626–639. 10.1166/jbn.2020.2914 32919483

[B33] Wang F. Shi Y. Lu L. Liu L. Cai Y. Zheng H. (2012). Targeted Delivery of GDNF through the Blood-Brain Barrier by MRI-Guided Focused Ultrasound. PLoS One 7 (12), e52925. 10.1371/journal.pone.0052925 23300823 PMC3531370

